# COVID-19 among Chronic Dialysis Patients after First Year of Pandemic, Argentina

**DOI:** 10.3201/eid2811.212597

**Published:** 2022-11

**Authors:** Augusto Vallejos, Andrea E.M. Baldani, Micaela A. Gauto, Dalila V. Rueda, Federico M. Santoro, Graciela Abriata

**Affiliations:** Dirección Nacional de Abordaje Integral de Enfermedades no Transmisibles, Buenos Aires, Argentina (A. Vallejos, G. Abriata);; Dirección Nacional de Epidemiología e Información Estratégica, Buenos Aires, Argentina (A.E.M. Baldani, M.A. Gauto, D.V. Rueda, F.M. Santoro).

**Keywords:** COVID-19, SARS-CoV-2, chronic renal insufficiency, renal dialysis, respiratory infections, zoonoses, epidemiology, coronavirus disease, viruses, severe acute respiratory syndrome coronavirus 2, Argentina

## Abstract

We performed a descriptive study to characterize effects from COVID-19 among chronic dialysis patients compared with the general population in Argentina during March 2020–February 2021. COVID-19 case-fatality rate of chronic dialysis patients was 10 times the national rate; the age-standardized mortality ratio was 6.8 (95% CI 6.3–7.3).

Patients requiring dialysis for chronic kidney disease comprise a high risk to public health (*1*), and need for this treatment precluded patients from being able to comply with COVID-19 isolation measures during the pandemic (*2*). Studies have reported high COVID-19 mortality rates among these patients, but such studies have been scarce in Latin America (*3*–*5*). We contrasted clinical and epidemiologic characteristics and outcomes between chronic dialysis (CD) patients and the general population to evaluate COVID-19 dynamics during the first year of the pandemic in Argentina. 

## The Study

We designed an observational, analytic, retrospective, nationwide study that included data from all COVID-19 cases reported to the National Health Surveillance System (SNVS^2.0^) during epidemiologic weeks (EW) 10/2020 (March 1–7, 2020) through 08/2021 (February 21­­–27, 2021). COVID-19 cases in CD patients included all cases in persons on dialysis treatment at the time of COVID-19 diagnosis. On March 1, 2021, we downloaded data from an SNVS^2.0^ database that included COVID-19 cases reported through EW 08/2021. Notifications provided demographic, clinical, and epidemiologic data; we validated cases involving CD patients with records from the network of local kidney health institutions of the National Program of Integral Approach to Renal Diseases (Programa Nacional de Abordaje Integral de Enfermedades Renales [PAIER]).

We used projections from the National Institute of Statistics and Census (Instituto Nacional de Estadística y Censos [INDEC]) for the population of Argentina (*6*) and the Argentine Registry of Chronic Dialysis (Registro Argentino de Diálisis Crónica [RADC]) for the population of CD patients (*2*). We performed a descriptive analysis of COVID-19 cases in CD patients and the general population during the first year of the pandemic. We included only data from complete records for each variable. For epidemiologic description in the temporal analysis, we determined EW dates on the basis of patient symptom onset or, if unavailable, sample collection. We classified cases as close-contact, community-acquired, or other according to epidemiologic history. 

We described age-group distribution for total and deceased case-patients for both populations. We also calculated cumulative incidence and overall and age-group case-fatality rates (CFR) and age-standardized incidence and mortality ratios by indirect adjustment method. We counted as deceased those persons recorded as having died in their SNVS^2.0^ notifications and the rest, including patients who had recovered or were active case-patients, as nondeceased. We did not include deaths that occurred after COVID-19 isolation and follow-up were completed. 

We calculated qualitative variables with frequency distributions and quantitative variables using median and interquartile range (IQR). We performed quantitative data analysis using Student t-test and tested difference in proportions using Z-test or Fisher exact test according to assumptions. We defined 2-sided p values <0.05 as statistically significant. We performed statistical analyses using RStudio version 1.2 18 software (https://www.rstudio.com). 

**Figure 1:**
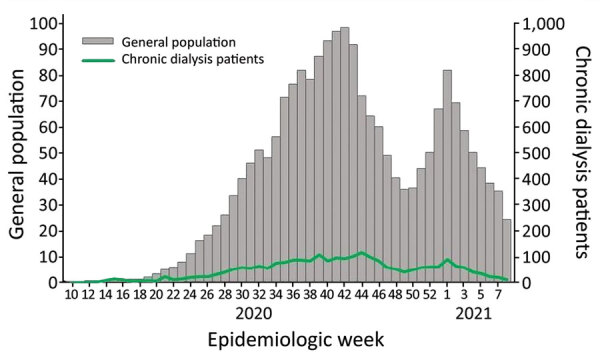
COVID-19 cases in the general population (per 1,000 persons) and chronic dialysis patients, by date of symptom onset, Argentina, epidemiological weeks 10/2020 (March 1–7, 2020) through 08/2021 (February 21–27, 2021).

During the study period, 2,107,676 people from the general population and 2,496 persons requiring CD were diagnosed with COVID-19 (Table). Cumulative incidence was 46 cases per 1,000 among the general population and 83/1,000 among CD patients. The epidemic curve for COVID-19 cases in the general population started during EW 10/2020; the first COVID-19 case in a CD patient was registered during EW 13/2020. Epidemic curves for both populations followed the same trends over time (Figure 1). 

**Table:** Characteristics of COVID-19 cases in the general population and in chronic dialysis patients, Argentina, 2020–2021

Characteristic	General population, n = 2,107,676	Chronic dialysis patients, n = 2,496
Sex, no. (%)		
F	1,045,989 (49.6)	1,076 (43.1)
M	1,036,211 (49.2)	1,419 (56.9)
Other	2,4631 (1.2)	1 (0.0)
Unknown	845 (0.0)	0 (0.0)
Median age, y (IQR)	37 (27–51)	60 (48–70)
Epidemiologic case classification, no. (%)		
Close-contact cases	310,041 (14.7)†	439 (17.6)
Community-acquired cases	1,546,887 (73.4)†	1,731 (69.3)
Other	249,712 (11.9)†	326 (13.1)
Deceased case-patients, no. (%)	52,075 (2.4)	617 (24.7)
Deceased case-patients median age, y (IQR)	73 (63–82)	67 (58–75)

*IQR, interquartile range. †Relative frequencies were calculated according to the cases with information on the variable. A total of 2,106,640 COVID-19 cases were contemplated in the general population.

Case distribution by age group showed higher proportions in older age groups among CD patients than the general population (Appendix) and a significantly higher median age among CD patients, 60.0 (IQR 48–70) years of age, than among the general population, 37.0 (IQR 27–51) years of age (p<0.05). When standardized by age, COVID-19 incidence in CD patients was 1.5 (95% CI 1.5–1.6) times the national rate. Case distribution by sex showed a slightly higher proportion of male case-patients among CD patients, although this difference was not significant (Table).

**Figure 2:**
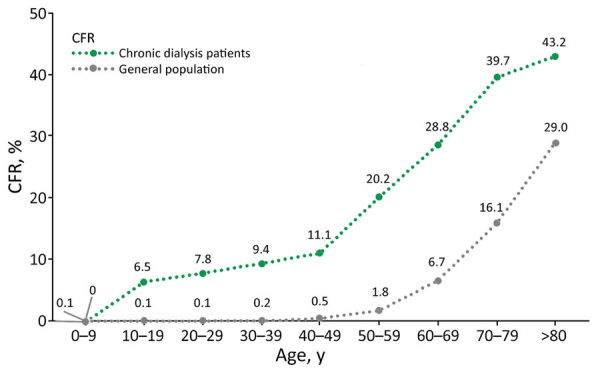
CFR for COVID-19 in the general population and chronic dialysis patients, by age group, Argentina, epidemiological weeks 10/2020 (March 1–7, 2020) through 08/2021 (February 21–27, 2021). CFR, case-fatality rate.

Deceased-case distribution was concentrated in older age groups among CD patients (Appendix). However, median age of death among CD patients was 67.0 (IQR 58–75) years of age, significantly lower than among the general population, 73.0 (IQR 63–82) years of age (p<0.05) (Table). There were 52,075 deaths among the general population (COVID-19 CFR 2.4%) and 617 among CD patients (COVID-19 CFR 24%) (Table); CFR among CD patients was significantly higher than for the general population among age groups 20–29 years and above (Figure 2). Age-standardized mortality ratio was 6.8 (95% CI 6.3–7.3). 

Most close-contact cases were recorded during the first weeks of the pandemic, after which community-acquired cases trended upward. After EW 15/2020, the percentage of close-contact cases was always higher among CD patients than national rates, and a statistically significant difference (p<0.05) was seen during EWs 15–20/2020 and EWs 35/2020–08/2021, the end of the study period (Appendix). Because hemodialysis is an outpatient treatment, patients must visit specialized centers several times a week to receive treatment, sometimes remaining in close proximity to other patients for several hours. In addition, carpooling to dialysis centers was common. Although we cannot rule out domestic exposure, dialysis modality presented a greater SARS-CoV-2 exposure risk (*2*). 

Analysis of COVID-19 dynamics for persons requiring CD during the first year of the pandemic in Argentina highlights the influence of conditions of vulnerability within an epidemiologic context. People with CD requirements tended to be older and more susceptible to infectious diseases. Requiring CD is associated with high mortality; the Argentine Registry of Chronic Dialysis reported that, of 30,300 CD patients in Argentina in 2019, 17% died (*1*). Temporal distribution of COVID-19 cases was similar in both groups. We observed ≈60% of cases among men, which correlates with the sex distribution among CD patients (*1*). National COVID-19 incidence among CD patients was twice that among the general population and 50% higher when adjusted by age. 

Although mortality rates vary among countries (*4*), COVID-19 CFR in CD patients (24.0%) is similar throughout Latin America; 1 study from Guatemala described a CFR of 27.7% (*3*). Compared rates for with the general population, CFR in CD patients was 10 times higher and exceeded national rates in all age groups. According to age-standardized mortality ratio, CD patients were 5.8 times as likely to die as predicted by national COVID-19 mortality trends.

Among limitations, our results were based on data obtained before national vaccination campaigns for this group. Although modality was not specified, 93.2% of dialysis patients in Argentina undergo chronic hemodialysis (*1*). In addition, we were unable to adjust mortality rates by underlying conditions because those conditions are self-reported nonmandatory information when reporting COVID-19 cases, resulting in incomplete data for that variable. 

## Conclusion

Our results show the substantial effect the first year of the COVID-19 pandemic had on CD patients in Argentina. These findings reinforce the importance of implementing prevention and control strategies and prioritizing vaccination campaigns among this population (*7*). 

Appendix Additional information about effects from COVID-19 among chronic dialysis patients in Argentina after the first year of the pandemic.
