# Supplementary material for: COVID-19 among Chronic Dialysis Patients after First Year of Pandemic, Argentina
Source: Emerg Infect Dis. 2022 Nov;28(11):2294–7. doi: 10.3201/eid2811.212597 (PMC9622233; doi:10.3201/eid2811.212597)
Supplement: Appendix — Additional information about effects from COVID-19 among chronic dialysis patients in Argentina after the first year of the pandemic. [file 21-2597-Techapp-s1.pdf]

Article DOI: https://doi.org/10.3201/eid2811.212597

COVID-19 among Chronic Dialysis Patients after First Year of Pandemic, Argentina

Appendix

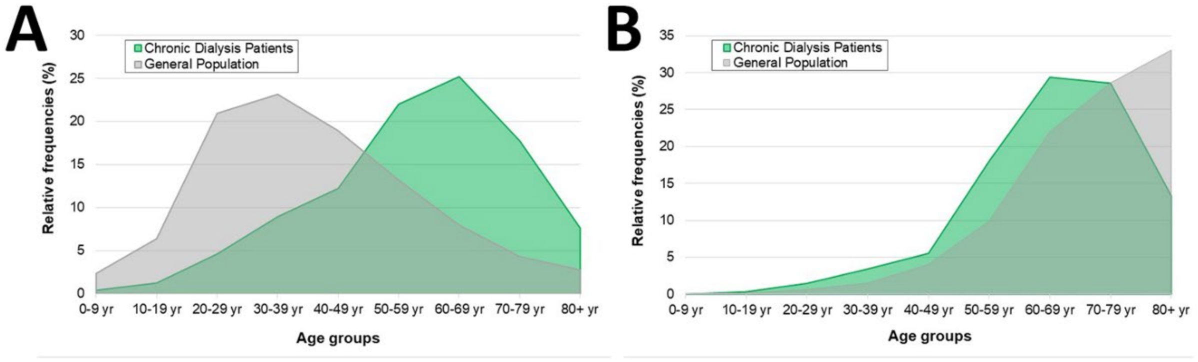

Appendix Figure 1. Relative frequencies of A) total COVID-19 cases (N = 2,082,160) and B) COVID-19 cases resulting in death (N = 50,297) among the general population and chronic dialysis patients in Argentina by age groups during epidemiologic weeks 10/2020–08/2021. We excluded 25,516 cases (1,778 deceased cases) among the general population and 1 (deceased) case among chronic dialysis patients because of incomplete data.

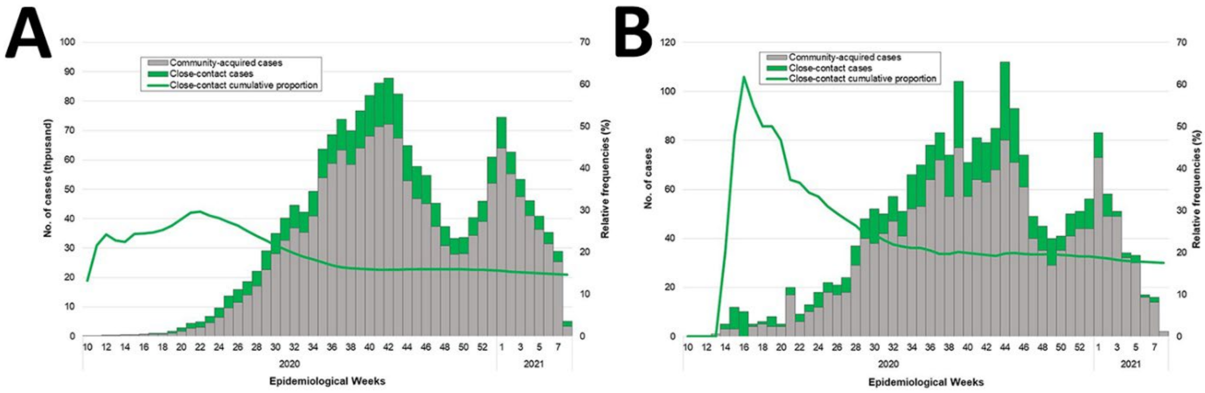

Appendix Figure 2. COVID-19 cases among A) the general population (N = 1,856,928) and B) chronic dialysis patients (n = 2,170) in Argentina by epidemiological week and case classification during epidemiological weeks 10/2020–08/2021. We excluded COVID-19 cases with the case-classification "other" from the figure.
